# Supplementary material for: Content-rich biological network constructed by mining PubMed abstracts
Source: BMC Bioinformatics. 2004 Oct 8;5:147. doi: 10.1186/1471-2105-5-147 (PMC528731; doi:10.1186/1471-2105-5-147)
Supplement: Additional File 2 — The original results of the above study (non-essential files are deleted to keep the file size under the limit set by BMC bioinformatics). [file 1471-2105-5-147-S2.bz2 › chilibotAdditionalFile2/dip05/412ID10712517E10078/html/BMPR1B_BMPR1A.html]

 


 **BMPR1B** and **BMPR1A** 
  
Found 25 abstracts in PubMed, retrieved 05.  
 

 What does Google say? 
 PDF only 
| .edu only 

---

**Interactive relationship** (e.g. stimulation, inhibition, etc)

**Non-interactive relationship** (e.g. studied together, co-existance, homology, etc.)

- We assessed the distribution and relative immunohistochemical staining intensity of the bone morphogenetic protein 7, osteogenic protein 1 OP 1, in its pro and mature forms, and four of its receptors, type I ALK 2, ALK 3  [ **BMPR1A** ] , and ALK 6  [ **BMPR1B** ]  and type II in normal adolescent New Zealand White rabbit articular cartilage.  Ref: 12364567 J Histochem Cytochem, 2002
- The sections were rehydrated, submitted to microwave antigen retrieval and treated with polyclonal antibodies against  **BMPR1A** ,  **BMPR1B**  and BMPR2.  Ref: 11882013 Reproduction, 2002
- A mechanistic explanation for increased pSmad1 5 staining during inflammation was provided by the upregulated expression of all the BMP type I receptors, i.e., activin receptor like kinase ALK 2, ALK3  [ **BMPR1A** ] , and ALK6  [ **BMPR1B** ] , in the inflamed bronchial epithelial cells.  Ref: 12151307 Am J Respir Cell Mol Biol, 2002
- Messenger RNAs for BMP, 7, the BMP type I receptors Act R 1 alk 2, activin like kinase receptor, BMPR IA alk 3  [ **BMPR1A** ] , IB alk 6  [ **BMPR1B** ] , and the type II receptor BMPR II were detected.  Ref: 11829442 Biomaterials, 2002
- Activin receptor like kinase ALK 1, ALK 2, ALK 3  [ **BMPR1A** ] , and ALK 6  [ **BMPR1B** ]  were able to reproduce the hypertrophic maturation of chondrocytes induced by BMP 2.  Ref: 12082094 J Biol Chem, 2002
